# Halloysite supplemented in the diet, and sex of Cherry Valley ducks affects growth, carcass composition, meat quality, and jejunum and leg bone strength

**DOI:** 10.1186/s12917-025-04764-2

**Published:** 2025-05-02

**Authors:** Sebastian Wlaźlak, Mirosław Banaszak, Jakub Biesek

**Affiliations:** Department of Animal Breeding and Nutrition, Faculty of Animal Breeding and Biology, Bydgoszcz University of Science and Technology, 85-084 Bydgoszcz, Poland

**Keywords:** Aluminosilicates, Waterfowl, Efficiency, Chemical composition

## Abstract

**Background:**

Natural minerals can be innovative feed additives in the waterfowl nutrition, affecting the production efficiency and the meat quality. The study assessed production results, carcass characteristics, meat quality, and strength of the jejunum and leg bones of male and female Cherry Valley ducks fed with 1% halloysite. The ducks were kept in control groups (males and females) and fed a commercial diet. In the experimental groups (males and females), 1% halloysite was added to the diet throughout the rearing period. 50 ducks in 5 repetitions were in each group. During 42 days of rearing, the ducks' body weight, growth, feed intake, and conversion ratio were controlled and calculated. After rearing, 10 carcasses per group were selected and dissected. The physicochemical characteristics of the leg and pectoral muscles and the strength of the jejunum and leg bones were analyzed.

**Results:**

The application of halloysite reduced the body weight of birds (*P* = 0.049) and body weight gain (*P* = 0.048) on day 42 and throughout the rearing period and increased the liver weight of ducks (*P* = 0.020). Female carcasses were characterized by a higher weight of pectoral muscle (*P* = 0.005), muscle total (*P* = 0.015), and abdominal fat (*P* = 0.007), and males by a higher weight of carcass remains (*P* = 0.013). In the pectoral muscles of ducks where the mineral was added, significantly lower protein content and higher collagen (*P* < 0.001), intramuscular fat (*P* < 0.001), and water (*P* = 0.014) content were found. The leg muscles of the birds from the control groups were characterized by significantly higher redness (*P* = 0.003) and yellowness (*P* = 0.031), and males had a higher content of intramuscular fat compared to females (*P *< 0.001). Halloysite increased the jejunum tensile strength (*P* = 0.023).

**Conclusions:**

Halloysite adversely impacted ducks' body weight and weight gain while altering meat quality by increasing pectoral muscle pH (*pectoralis major*) and fat content (*pectoralis major* and *pectoralis minor*) and changing leg muscles' color. Jejunum tensile strength was higher post-halloysite supplementation. These results suggest halloysite has both positive and negative effects on duck growth, meat properties, and jejunum strength, warranting further research.

## Background

The world's duck meat production is dominated by Asian countries, mainly China, Vietnam, Malaysia, and Indonesia [1]. Over the last few years, production in Europe has decreased from 555,000 tonnes of carcass weight in 2018 to 445,000 tonnes in 2022. Poland is the only country in the EU where this sector has recorded the most intense growth—by over 50% [2]. The dynamic increase in production volumes requires optimization in nutrition, welfare, and flock management to utilize the genetic potential of commonly used hybrids fully. In addition, better feed efficiency and low disease incidence are critical aspects that will ensure the stable development of this industry sector [3]. Currently, commercial hybrids of Pekin-type ducks are most widely used in global production [4]. They are characterized by a short rearing period, good body weight gain, high feed efficiency, and resistance to unfavorable environmental conditions [5].

High quality and safety of poultry meat are the two most important aspects of sustainable production [6]. The European Union banned antibiotic growth promoters in poultry production in 2006 [7]. Thus, investigating natural alternatives to improve production efficiency, profitability, and poultry health began [8–10]. The studies concerned probiotics, prebiotics, synbiotics, herbs, enzymes, organic acids, and natural minerals [11–16]. The validity of their use in broiler chickens, ducks, and laying hens was verified [17], and some of them can be administered using various methods [18]. This trend has also led to the reduction of the use of antibiotic veterinary drugs in favor of other agents that do not harm the natural environment. It highly fits into currently implemented food production strategies [19].

Halloysite is a natural clay mineral with a micro- and nanotube structure and a solid similarity to kaolin. Its natural deposits occur in Europe, Asia, and South America [20]. High porosity and lack of cytotoxic effects contributed to the search for various possibilities of its use, e.g., encapsulation of biologically active molecules or nanofiller [21]. Due to its specific properties, research has been conducted on its protective effect against mycotoxins in rats (in vitro) [22] and also in pig feeding [23]. The cited authors demonstrated high effectiveness in reducing the negative impact of zearalenone by halloysite, especially after modification. Halloysite was also used as an additive in the nutrition of broiler chickens and laying hens [24, 25].

As shown by Nadziakiewicz et al. [26], halloysite at the level of 1% in the feed for broiler chickens positively affected their carcass yield and the levels of triglycerides, total cholesterol, and LDL fraction in blood serum. The authors point out that the positive effect on carcass yield could have resulted from increased digesta retention time and better endogenous enzyme action. A previous study confirmed improvement of feed conversion ratio and better protein utilization when halloysite was used in feed for broiler chickens [25]. Due to its adsorption properties, halloysite may benefit the bedding quality or reduce *footpad dermatitis* in Ross 308 broiler chickens [27]. The mixture of halloysite and vermiculite in the bedding during turkey rearing reduced ammonia emissions by 15% and volatile organic substances by 13–83% [28].

Moreover, it may modify broiler chickens'protein content in the pectoral and leg muscles due to increased intestinal villi height and better absorption of nutrients from feed [29]. Higher growth and improvement of selected meat quality parameters of broiler chickens (pectoral muscle weight or water-holding capacity in pectoral muscles) were found after using a mixture of halloysite and zeolite in feed and pellet bedding [29]. Chung and Choi [30] found no significant changes in body weight and weight gain after using 2% bentonite and 2% ilite in broiler duck feeding. However, the authors showed a significantly higher total nitrogen content in the litter, which is beneficial for further bedding management. In the study of Kook et al. [31], 1.5% felspar-illite (felspar) did not affect production results but significantly reduced the fat content of meat compared to the control group. In a pilot study [32], 4% zeolite was added to the diet of Cherry Valley and Orvia commercial crossbred broiler ducks. Duck body weight gain decreased, and poor feed conversion ratio and higher water-holding capacity in the pectoral muscles were demonstrated. Therefore, the effect of aluminosilicate needed to be clarified.

Most natural minerals were mainly subject to research on the impact of their use on the bedding, production results, carcass composition, and meat quality of broiler chickens. There is limited literature on the use of halloysite in duck feeding. Therefore, research was undertaken on the impact of adding halloysite in feed for broiler ducks, which allowed for expanding the available knowledge in this area and verifying the hypothesis, which is as follows: The 1% halloysite supplementation in feed for both sexes Cherry Valley broiler ducks has an impact on the growth performance, carcass and meat quantitative and qualitative features, and physical characteristics of the jejunum and leg bones. The study aimed to evaluate the production results, carcass characteristics, meat quality, and strength of the jejunum and leg bones of Cherry Valley ducks fed with 1% halloysite supplementation.

## Methods

### Animals and diet

In the experiment, 200 one-day-old Cherry Valley SM3 Medium commercial hybrid ducklings were used. The ducklings came from a commercial waterfowl hatchery in central Poland (Tulce, Poland). The body weight of birds on day 1 was in the range of 57.55–59.50 g (SEM – 0.360). One hundred females and one hundred males were divided into 4 equal-sized groups (50 birds of each sex per group). The sex of ducks was included due to the potential differences between males and females, as it was shown by Kaewtapee et al. [33] when assessing the growth, production performance, and carcass quality of Cherry Valley ducks in both sexes. There was a control group of males (C × M) and females (C × F) and an experimental group of males (H × M) and females (H × F). Each group was kept in 5 replicates—pens (10 ducks for each). Rearing lasted 42 days and was divided into 2 feeding periods. The first one lasted from day 1 to 28, using a starter commercial diet. The second one lasted from day 29 to 42, and a grower commercial diet was used.

In all groups, the basis of nutrition was a commercial diet (De Heus, Ede, Netherlands) for broiler ducks with an appropriately balanced composition following the nutritional recommendations of poultry feed [34]. The experimental diets were isocaloric and isonitrogenous. The automatic drinkers were installed so that there were 2 nipple drinkers in each pen. The experimental groups (male and female) were fed with feed containing 1% halloysite throughout the rearing period. Halloysite came from a mine in southern Poland (Krotoszyce, Poland). The halloysite used in the experiment was characterized by the following physical properties: dusty structure, specific surface area of 65–86 m^2^/kg, and bulk density of 0.70–0.85 g/cm^3^. The chemical composition includes aluminum (13.00%), silicon (12.00%), calcium (0.40%), magnesium (0.30%), sodium (0.10%), potassium (0.08%), phosphorus (0.30%), iron (9.00%), titanium (1.00%), and manganese (0.20%). The starter and grower feeds were thoroughly mixed with halloysite to ensure thorough distribution. The area of the pens was 2 m^2^. The stocking density did not exceed 17 kg/m^2^. Chopped wheat straw was used as bedding. On the first day, the temperature in the duck house was 26 °C, which was gradually lowered to 18 °C in the 4 th week of the birds'life. An additional heat source in the form of a heating lamp was used, which maintained the temperature at 30 °C. Air humidity was 60–70%, and air movement did not exceed 0.2–0.3 m/s. The concentration of harmful gases was within the permissible levels, i.e., 3000 ppm – CO_2_, 20 ppm – NH_3_, and 5 ppm – H_2_S [35].

### Diet composition

Starter and grower commercial diets from the control and experimental groups were collected (about 500 g) in 5 bags of each type (20 samples in total). The analysis of the nutrient composition was consistent with the methods of the Polish Committee for Standardization (www.pkn.pl). The following ingredients were analyzed in the commercial diet and halloysite diet: dry matter [36], crude ash [37], crude protein [38], crude fiber [39], crude fat [40], neutral detergent fiber [41], acid detergent fiber, and acid-detergent lignin [42], starch [43]. Gross energy (GE) was determined following the PN-EN ISO 9831:2005 standards [44]. The KL-21 PLUS isoparabolic calorimeter (Precyzja-Bit PPHU LCC, Bydgoszcz, Poland) was used. All analyses were performed in 10 replicates (Table 1).

**Table 1:** Chemical composition of starter and grower feed for broiler ducks and halloysite

Nutrient^a^	Group		SEM
	Control	Halloysite (1%)	
	Starter diet (days 1–28)		
Gross energy (MJ/kg)	16.72	16.46	0.514
Dry matter (g/kg feed)	889.50	886.30	0.460
Crude ash (g/kg DM)	58.42	61.24	0.844
Crude protein (g/kg DM)	221.55	215.32	1.282
Crude fat (g/kg DM)	41.51	36.51	0.619
Crude fiber (g/kg DM)	54.78	56.84	1.651
ADF (g/kg DM)	55.22	53.33	1.423
NDF (g/kg DM)	138.77	144.64	1.442
ADL (g/kg DM)	22.74	26.58	0.697
Starch (g/kg DM)	414.41	437.20	3.281
	Grower diet (days 29–42)		
Gross energy (MJ/kg)	16.84	16.67	0.024
Dry matter (g/kg feed)	884.60	879.22	1.004
Crude ash (g/kg DM)	57.29	58.10	0.346
Crude protein (g/kg DM)	223.30	204.39	2.235
Crude fat (g/kg DM)	42.74	36.36	0.775
Crude fiber (g/kg DM)	52.01	52.70	0.259
ADF (g/kg DM)	49.77	57.73	1.241
NDF (g/kg DM)	141.91	150.14	1.247
ADL (g/kg DM)	24.45	24.36	0.259
Starch (g/kg DM)	426.19	440.30	1.902

Declared nutrient composition of a commercial diet

Starter feed: crude protein-19,5%, ether extract-3,9%; crude fiber-4,2%; lysine-0,93%; methionine-0,42%; threonine-0,72%; calcium-0,85%; phosphorus-0,69%; sodium-0,17%; vitamin A-10000 j. m.; vitamin D3-3000 j. m.; vitamin E-25 j. m.; Grower feed: crude protein-17,1%, ether extract-3,7%; crude fiber-4,5%; lysine-0,87%; methionine-0,37%; threonine-0,61%; calcium-0,81%; phosphorus-0,66%; sodium-0,16%; vitamin A-10000 j. m.; vitamin D3-3000 j. m.; vitamin E-25 j. m

^a^*DM* dry matter, *ADF* acid detergent fiber, *NDF* neutral detergent fiber, *ADL* acid detergent lignin

### Growth performance

Ducks were weighed on days 1, 28, and 42 (Radwag, Radom, Poland). Feed intake (FI) and deaths were recorded daily. Based on data, the body weight gain, feed conversion ratio, viability, European Production Efficiency Factor were calculated.

$$BWG=final body weight \left(g\right)-initial body weight (g)$$

$$FCR=\frac{feed intake \left(kg\right)}{body weight gain \left(kg\right)}$$

$$EPEF=\frac{viability \left(\%\right) x BW (kg)}{\begin{array}{c}age \left(days\right) x FCR \left(\frac{kg feed}{kg gain}\right)\end{array}}\times 100$$

### Slaughter and sample collection

On the last day of rearing (42nd), 10 ducks from each group with a body weight similar to the average body weight per pen were selected for slaughter. Stunning was performed using electric current [45]. The birds were then slaughtered by cutting the spinal cord between the first cervical vertebra and the occipital condyle. After bleeding, the carcasses were scalded in water at 65 °C for about 10 s and plucked using a mechanical plucker (Soda Pluss, Krepice, Poland). Then, the carcasses were covered with wax (Polwax, Jasło, Poland), approved for contact with food, to remove feather remnants after plucking. After removing the wax, the paws were cut off at the hocks, the carcasses were gutted, and the offal was isolated, i.e., the heart, liver, and gizzard. A 10 cm long jejunum was taken from each carcass (the reference point was Meckel’s diverticulum). The cooled carcasses and offal were placed in a refrigerator (Hendi, Poznań, Poland) at 4 °C for 24 h until meat quality analyses were conducted. The jejunum samples were frozen at −18 °C.

### Carcass features and meat quality

Carcasses and offal were weighed (Radwag, Radom, Poland). Carcasses were dissected by separating the neck, skin from the neck, pectoral muscles (*m. pectoralis major and minor*), leg muscles (thighs and drumsticks; deboned), skin with subcutaneous fat, wings with skin, abdominal fat, and carcass remains (trunk, leg bones). All elements were weighed (Radwag, Radom, Poland). The tibia and femur from the right leg of each carcass were packed in string bags and frozen (−18 °C) (Gorenje, Velenje, Slovenia) for subsequent strength analyses. Slaughter yield ($$slaughter yield=\frac{carcass weight \left(g\right)}{live body weight \left(g\right)}x 100$$%) and elements share in carcass were calculated [46].

The pH of the meat was measured using a pH meter with a dagger electrode (Elmetron, Zabrze, Poland) 24 h after slaughter. The measurement was performed in the right pectoral muscle. Before the analyses, the device was calibrated (Elmetron, Zabrze, Poland) in standard solutions with pH 4.00, 7.00, and 9.00. The color of the meat (24 h after slaughter) was measured with a CR-400 colorimeter (Konica Minolta, Tokyo, Japan) on a scale (CIE Lab): L*, a*, and b*, which means lightness, redness, and yellowness, respectively. The measurement was taken from the inside of the pectoral muscle (*pectoralis major*) and leg muscles. One pectoral muscle from each carcass was weighed for drip loss analyses (24 h after slaughter). Then, they were placed in a closed string bag measuring 150 × 200 mm and in a larger one measuring 150 × 250 mm. The smaller bag was slit at the bottom to allow water to leak out. The samples prepared this way were hung approximately 5 cm from each other and moved to a refrigerator at 4 °C (Hendi, Poznań, Poland). After 24 h, the muscles were weighed again, and drip loss was calculated ($100-\left(\frac{M2}{M1}\right)x 100)$, where M2 – muscle weight after 24 h, M1 – initial muscle weight. For water-holding capacity (WHC) analysis, each group's pectoral and leg muscles were ground in a meat grinder (Hendi, Poznań, Poland). Then, a sample of 0.300 g (± 0.005 g) was weighed and placed on a piece of Whatman 1 tissue paper, covered with another piece of tissue paper, and loaded with a 2 kg weight for 5 min. The meat remains were scraped off the paper and weighed. The obtained results allowed for the calculation of the WHC of the pectoral and leg muscles ($100-\left(\frac{M2}{M1}\right)x 100)$, where M2 – sample weight after 5 min, M1 – initial sample weight. The analyses were performed in 10 repetitions [47].

Approximately 90 g of previously ground pectoral and leg muscles were weighed for chemical analyses. The content of protein, intramuscular fat (IMF), collagen, salt (Cl-based), and water were verified with a FoodScan device (FOSS, Hillerød, Denmark) using near-infrared transmission spectrophotometry (NIT) [48]. The device has been calibrated to analyze the chemical composition of meat and meat products.

### Bones’ breaking strength and jejunum tensile strength

The previously frozen leg bones (tibia and femur) and jejunum were thawed at 4 °C for 24 h. An Instron 3345 device (Instron, Buckinghamshire, UK) with Bluehill 3 software was used. The tibia and femur bones were thoroughly cleaned of meat residues and weighed (Radwag WTC 2000, Radom, Poland). Each bone was placed on the Instron Bend Fixture 10 mm bone strength analyzer. The maximum force necessary to break the bone (N) was measured, and the force per 1 g of bone was calculated. The measurement speed was 250 mm/min. The jejunum's tensile strength analysis was stretching and verifying the higher force necessary to tensile the intestine (N). The strength was analyzed using the Instron Pneumatic Grip 2 kN. The measurement speed was 500 mm/min [32].

### Statistical analyzes

The data were prepared using the statistical program Statistica, ver. 13.3.0 (TIBCO, Software, Kraków, Poland, 2017). Mean values and standard error of the mean (SEM) were calculated for each feature. Normality of distribution test (Kolmogorov–Smirnov test) and homogeneity of variance test (Levene test) were performed. A one-way analysis of variance (ANOVA) was used for statistical calculations. For the main effects (halloysite supplementation or sex of ducks), an appropriate statistical model was used: Yh/s = µ + Ch/s + eh/s, where Yh/s – the dependent variable; µ – the overall mean; Ch/s – group: Control and Halloysite or Male and Female, and eh/s – residual error. The interaction between main factors (halloysite supplementation and sex of ducks) was also analyzed by two-way analysis of variance with statistical model: Y_HS_ = µ + C_H_ + D_S_ + CD_HS_ + e_HS_, where Y_H/S_, the dependent variable; µ, the overall mean; C_H_, the effect of halloysite supplementation (Control, Halloysite); D_S_, the impact of sex of ducks (Male, Female); CD_HS_, the interaction between H and S; e_HS_, residual error). The statistical significance of differences was verified using the Tukey test with a probability of P ≤ 0.05. Tukey's test was standardly chosen to verify statistically significant differences. In the case of jejunum tensile strength, an additional analysis was performed using a less conservative NIR test (Fisher's Least Significant Difference—LSD) than Tukey's test. Pearson correlation coefficients (r) were calculated between qualitative physicochemical features of the pectoral and leg muscles, taking into account P-value < 0.05. Negative r values indicate a negative dependence between the features, and positive r values—positive ones. Values of r, correlation: < 0.2 – no correlation between variables; 0.2–0.4 – weak correlation; 0.4–0.7 – moderate correlation; 0.7–0.9 – quite strong correlation; > 0.9 – very strong correlation.

## Results

### Growth performance

A higher BW of ducks on day 28 in the control group than in the halloysite group was found (*P* = 0.001). Similarly, statistical differences were found in BW on day 42 (*P* = 0.049) and BWG for the whole rearing period (*P* = 0.048). In the first feeding period (days from 1 to 28), the higher FCR in the experimental group, where ducks were fed with 1% of halloysite, was noticed (*P* = 0.003). These results described the main effect of halloysite addition. Analysis of sex influence has shown no statistical differences in production results (*P* > 0.05). The interaction of these two main factors was also calculated. On day 28, BW in the control group of females (C × F) was higher compared to the halloysite group, both sexes (*P* = 0.005). The same differences in BWG from day 1 to day 28 were found (*P* = 0.005). The higher FCR in the first rearing period (days 1–28) in the group of males fed with 1% of halloysite (H × M) was found when compared to males from the control group (C × M) (*P* = 0.024) (Table 2).

**Table 2:** Growth performance of broiler ducks fed diets supplemented with 1% halloysite

	Item^1^* n* = 5 Groups^2^	Viability (%)	BW (g/bird)			BWG (g/bird)			FI (g/bird)			FCR (kg/kg)			EPEF
			Day 1	Day 28	Day 42	Days 1–28	Days 29–42	Total	Days 1–28	Days 29–42	Total	Days 1–28	Days 29–42	Total	
Main effects	Control	98.00	58.47	1966.26^a^	3090.61^a^	1907.79^a^	1124.35	3032.14^a^	3886.77	4294.12	7475.31	2.04^b^	3.85	2.47	292.48
	Halloysite	96.00	57.97	1845.25^b^	2969.55^b^	1787.28^b^	1124.30	2911.58^b^	3887.54	4178.20	7369.76	2.18^a^	3.74	2.53	269.34
	P-value	0.355	0.502	0.001	0.049	0.001	0.999	0.048	0.991	0.301	0.500	0.003	0.572	0.252	0.055
	Male	96.00	58.65	1889.79	3055.80	1831.14	1166.01	2997.15	3865.01	4247.92	7450.80	2.12	3.66	2.49	282.16
	Female	98.00	57.80	1921.72	3004.36	1863.92	1082.63	2946.56	3909.30	4224.39	7394.27	2.10	3.93	2.51	279.67
	P-value	0.355	0.251	0.453	0.426	0.441	0.076	0.432	0.526	0.836	0.719	0.760	0.146	0.730	0.844
Interaction	C × M	98.00	59.40	1929.65^ab^	3115.49	1870.25^ab^	1185.84	3056.09	3777.11	4254.86	7378.07	2.02^b^	3.60	2.42	334.22
	C × F	98.00	57.55	2002.87^a^	3065.73	1945.32^a^	1062.86	3008.18	3996.43	4333.37	7572.55	2.05^ab^	4.10	2.52	316.28
	H × M	94.00	57.89	1849.93^b^	2996.11	1792.03^b^	1146.18	2938.22	3952.90	4240.98	7523.54	2.21^a^	3.73	2.56	299.16
	H × F	98.00	58.05	1840.58^b^	2942.99	1782.53^b^	1102.41	2884.93	3822.18	4115.41	7215.99	2.15^ab^	3.76	2.50	305.43
	P-value	0.468	0.296	0.005	0.225	0.005	0.295	0.226	0.050	0.599	0.358	0.024	0.254	0.336	0.185
	SEM	1.051	0.360	20.576	31.275	20.596	23.595	31.148	33.723	54.644	75.667	0.019	0.075	0.023	6.107

^1^*BW* body weight, *BWG* body weight gain, *FI* feed intake, *FCR* feed conversion ratio, *EPEF* European Production Efficiency Factor; ^2^, C × M, male control group; C × F, female control group; H × M, the experimental male group fed with 1% halloysite; H × F, the experimental female group fed with 1% halloysite

^a,^^b^mean values with various letters in the row differ statistically significantly between all groups, considering *P* < 0.05

### Carcass composition

The application of halloysite significantly increased birds'liver weight (*P* = 0.020). Males were characterized by significantly higher gizzard weight (*P* = 0.004), and carcass remains (*P* = 0.013), and females had higher pectoral muscle weight (*P* = 0.005), total muscle weight (*P* = 0.015), and abdominal fat weight (*P* = 0.007). In males fed with halloysite, significantly higher liver weight was found compared to birds in the control groups (*P* = 0.018), and gizzard weight in both groups of males compared to females in the control group (*P* = 0.044). Significantly lower pectoral muscles (*P* = 0.009) and leg muscles (*P* = 0.049) weight were found in males fed with halloysite feed compared to females of both groups. Statistically, significantly higher abdominal fat weight in ducks with halloysite compared to drakes with halloysite (*P* = 0.004), and carcass remains in drakes with halloysite compared to females in control (*P* = 0.049) were found (Table 3).

**Table 3:** The carcass composition of broiler ducks fed diets supplemented with 1% halloysite

Item, *n* = 10		%		g/100 g carcass with offals			g/100 g carcass								
Groups^1^		Slaughter yield	Slaughter yield with offal	Heart	Liver	Gizzard	Neck	Pectoral muscle	Leg muscle	Muscles total	Skin with subcutaneous fat	Abdominal fat	Fat total	Wings with skin	Carcass remains
Main effects	Control	67.21	68.85	0.82	3.69^b^	4.29	7.75	18.16	14.12	32.28	19.64	0.60	20.24	13.05	26.69
	Halloysite	66.87	68.50	0.81	4.13^a^	4.30	7.91	17.04	14.64	31.68	18.68	0.58	19.27	13.27	27.88
	P-value	0.652	0.639	0.848	0.020	0.960	0.669	0.110	0.295	0.469	0.151	0.822	0.169	0.544	0.214
	Male	66.62	68.27	0.83	4.03	4.53^a^	7.96	16.65^b^	14.35	31.00^b^	18.75	0.49^b^	19.24	13.36	28.44^a^
	Female	67.46	69.08	0.80	3.79	4.06^b^	7.70	18.55^a^	14.41	32.96^a^	19.57	0.69^a^	20.26	12.95	26.13^b^
	P-value	0.260	0.284	0.307	0.221	0.004	0.471	0.005	0.902	0.015	0.219	0.007	0.145	0.255	0.013
Interaction	C × M	66.56	68.23	0.84	3.65^b^	4.55^a^	7.82	17.53^ab^	14.23	31.77^ab^	19.05	0.58^ab^	19.64	13.07	27.71^ab^
	C × F	67.86	69.48	0.80	3.73^b^	4.03^b^	7.69	18.79^a^	14.00	32.79^a^	20.22	0.61^ab^	20.84	13.03	25.66^b^
	H × M	66.68	68.31	0.82	4.41^a^	4.52^a^	8.11	15.77^b^	14.46	30.23^b^	18.45	0.39^b^	18.84	13.66	29.16^a^
	H × F	67.06	68.68	0.81	3.86^ab^	4.08^ab^	7.71	18.30^a^	14.82	33.12^a^	18.92	0.77^a^	19.69	12.88	26.60^ab^
	P-value	0.610	0.644	0.677	0.018	0.044	0.845	0.009	0.701	0.049	0.277	0.004	0.253	0.438	0.049
	SEM	0.368	0.373	0.013	0.097	0.086	0.182	0.352	0.247	0.409	0.329	0.040	0.350	0.178	0.475

^1^*C* × *M* male control group, *C* × *F* female control group, *H* × *M* the experimental male group fed with 1% halloysite, *H* × *F* the experimental female group fed with 1% halloysite

^a,^^b^mean values with various letters in the row differ statistically significantly between all groups, considering* P* < 0.05

### Physiochemical properties of pectoral and leg muscle

The use of halloysite significantly increased pH (*P* = 0.037), collagen (*P* < 0.001), intramuscular fat (IMF) (*P* < 0.001), and water content (*P* = 0.014) and reduced the protein content (*P* = 0.037) in the pectoral muscles compared to the control groups. In the pectoral muscles of females, a significantly lower IMF (*P* < 0.001), a higher protein (*P* = 0.011), and salt content (*P* = 0.009) were found compared to males. In the meat of drakes fed with halloysite, the lowest content of protein, the highest collagen (*P* < 0.001), and intramuscular fat content (*P* < 0.001) compared to the other groups were found. The meat of drakes from the control group was characterized by a significantly lower content of salt in the other groups of females (*P *= 0.006) and water content in all other groups (*P* < 0.001) (Table 4).

**Table 4:** Physicochemical properties of ducks’ pectoral muscle fed diets supplemented with 1% halloysite

Item^1^ *n* = 10						g/100 g muscle						
Group^2^		pH _24 h_	L*	a*	b*	Drip loss	WHC	Protein	Collagen	Salt	IMF	Water
Main effects	Control	5.92^b^	41.75	14.98	2.39	1.08	33.06	21.95^a^	1.28^b^	0.23	1.12^b^	75.80^b^
	Halloysite	6.04^a^	42.16	14.84	2.39	1.28	32.02	21.81^b^	1.41^a^	0.26	1.56^a^	75.96^a^
	P-value	0.037	0.684	0.809	0.990	0.348	0.369	0.037	< 0.001	0.271	< 0.001	0.014
	Male	5.97	41.94	14.87	2.32	1.25	31.84	21.80^b^	1.37	0.22^b^	1.52^a^	75.83
	Female	5.98	41.97	14.95	2.46	1.11	33.24	21.96^a^	1.32	0.28^a^	1.16^b^	75.93
	P-value	0.855	0.977	0.884	0.733	0.513	0.218	0.011	0.106	0.009	< 0.001	0.143
Interaction	C × M	5.92	41.56	14.93	2.38	0.97	32.11	21.95^a^	1.25^c^	0.18^b^	1.23^b^	75.64^b^
	C × F	5.92	41.94	15.04	2.40	1.20	34.00	21.94^a^	1.30^bc^	0.29^a^	1.02^c^	75.97^a^
	H × M	6.03	42.32	14.81	2.27	1.53	31.56	21.65^b^	1.49^a^	0.26^ab^	1.82^a^	76.03^a^
	H × F	6.04	42.00	14.87	2.52	1.03	32.49	21.98^a^	1.33^b^	0.27^a^	1.30^b^	75.89^a^
	P-value	0.231	0.964	0.994	0.979	0.200	0.481	< 0.001	< 0.001	0.006	< 0.001	< 0.001
	SEM	1.359	0.498	0.293	0.200	0.101	0.566	0.033	0.017	0.013	0.054	0.032

^1^L*, lightness; a*, redness; b*, yellowness, WHC, water holding capacity; IMF, intramuscular fat; ^2^, C × M, male control group; C × F, female control group; H × M, the experimental male group fed with 1% halloysite; H × F, the experimental female group fed with 1% halloysite

^a^^,b…^, mean values with various letters in the row differ statistically significantly between all groups, considering* P *< 0.05

Pearson's correlation between physicochemical features of the pectoral and leg muscles was analyzed. A negative, quite strong correlation was found for lightness and redness of pectoral muscle at a significance level of *P* < 0.001. A weak negative correlation for drip loss and protein content was found (P = 0.013), and a negative moderate correlation for protein and collagen (*P* < 0.001), intramuscular fat (*P* < 0.001), and water (*P* = 0.003) were found. A statistically significantly positive correlation was found in the case of collagen and salt content (weak), intramuscular fat, and water (moderate) (Table 5).

**Table 5:**
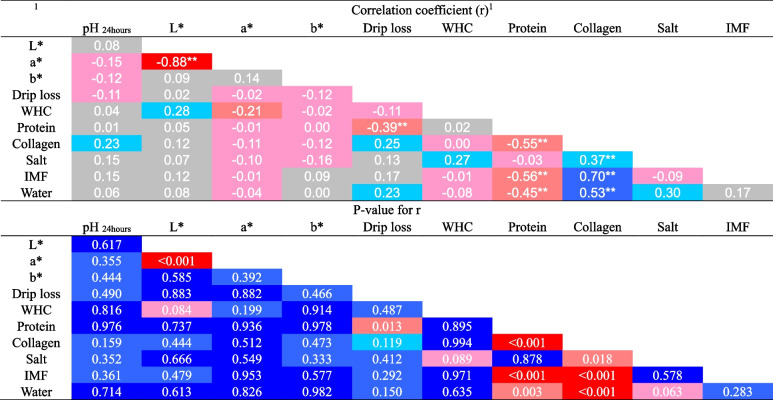
Pearson’s correlation between physicochemical features of the pectoral muscle

^1^L*, lightness; a*, redness; b*, yellowness; WHC, water holding capacity; IMF, intramuscular fat; negative values ​​- negative correlation; positive values ​​- positive correlation; values of r, correlation: <0.2 – no correlation between variables; 0.2–0.4 – weak correlation; 0.4–0.7 – moderate correlation; 0.7–0.9 – quite strong correlation; >0.9 – very strong correlation; ^**^, statistically significant correlation coefficient, *P*-value <0.05

The leg muscles of ducks fed with halloysite were characterized by significantly lower redness (*P *= 0.003) and yellowness (*P* = 0.031) compared to the control. In females, significantly higher collagen (*P* = 0.007) and salt content (*P* < 0.001) were found, and a lower IMF content (*P* < 0.001) compared to males was found. In the male leg muscles from the control group, higher redness and (*P* = 0.012) yellowness compared to males fed with halloysite (*P* = 0.040) and a higher IMF content compared to the other groups (*P* < 0.001) were shown. On the other hand, the female leg muscles fed mineral had significantly more collagen compared to males without the additive (*P* < 0.001) and males with the addition of mineral (*P* < 0.001) (Table 6).

**Table 6:** Physicochemical properties of ducks’ leg muscle fed diets supplemented with 1% halloysite

Item^1^ *n* = 10					g/100 g muscle					
Group^2^		L*	a*	b*	WHC	Protein	Collagen	Salt	IMF	Water
Main effects	Control	40.00	15.36^a^	3.56^a^	27.86	19.71	1.53	0.58	4.60	74.01
	Halloysite	39.16	13.39^b^	2.48^b^	29.52	19.62	1.61	0.56	4.32	73.53
	P-value	0.433	0.003	0.031	0.110	0.306	0.352	0.554	0.140	0.482
	Male	39.93	14.31	3.02	28.05	19.66	1.46^b^	0.52^b^	4.81^a^	73.87
	Female	39.23	14.43	3.02	29.33	19.68	1.68^a^	0.62^a^	4.11^b^	73.67
	P-value	0.512	0.862	0.993	0.221	0.862	0.007	< 0.001	< 0.001	0.771
Interaction	C × M	40.57	15.77^a^	3.97^a^	26.6	19.56^bc^	1.41^b^	0.55^bc^	5.34^a^	74.08
	C × F	39.44	14.94^ab^	3.16^ab^	29.11	19.87^a^	1.66^ab^	0.61^ab^	3.85^c^	73.94
	H × M	39.3	12.85^b^	2.08^b^	29.49	19.76^b^	1.51^ab^	0.50^c^	4.27^b^	73.66
	H × F	39.02	13.93^ab^	2.88^ab^	29.55	19.49^c^	1.71^a^	0.63^a^	4.36^b^	73.41
	P-value	0.758	0.012	0.040	0.136	0.002	0.041	< 0.001	< 0.001	0.903
	SEM	0.529	0.345	0.254	0.519	0.042	0.043	0.012	0.094	0.334

^1^L*, lightness; a*, redness; b*, yellowness, WHC, water holding capacity; IMF, intramuscular fat; ^2^, C × M, male control group; C × F, female control group; H × M, the experimental male group fed with 1% halloysite; H × F, the experimental female group fed with 1% halloysite

^a^^,b^mean values with various letters in the row differ statistically significantly between all groups, considering *P* < 0.05

In the leg muscles, a statistically significantly positive weak correlation was found for lightness and yellowness (*P* = 0.022), WHC and protein content (*P* = 0.031), and collagen and salt content (*P* = 0.015). A moderate positive correlation was found between redness and yellowness (*P* < 0.001) and protein and water content (*P* < 0.001). Significantly negative correlations were found for lightness and collagen content (moderate), protein and IMF content (moderate), and collagen and IMF (weak) (Table 7).

**Table 7:**
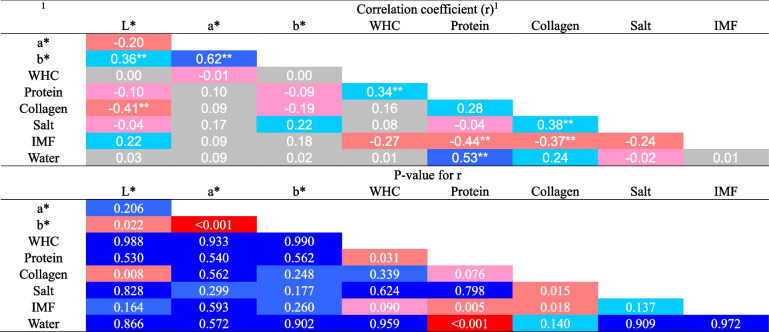
Pearson’s correlation between physicochemical features of the leg muscle

^1^L*, lightness; a*, redness; b*, yellowness; WHC, water holding capacity; IMF, intramuscular fat;negative values ​​- negative correlation; positive values ​​- positive correlation; values of r, correlation: <0.2 – no correlation between variables; 0.2–0.4 – weak correlation; 0.4–0.7 – moderate correlation; 0.7–0.9 – quite strong correlation; >0.9 – very strong correlation; ^**^, statistically significant correlation coefficient, *P*-value <0.05

### Jejunum tensile strength and bones breaking strength

Statistically, significantly heavier femur bones were found in females fed with halloysite compared to males fed with halloysite and females in the control group (*P* < 0.001). The jejunum of the birds fed with halloysite was characterized by significantly higher tensile strength than the control (*P* = 0.023). In the case of males from the control group, significantly lower tensile strength of the jejunum was shown compared to females from the control group (*P* = 0.025) (Table 8).

**Table 8:** Ducks’ leg bones weight and breaking strength and jejunum tensile strength fed diets supplemented with 1% halloysite

Item* n* = 10		Tibia			Femur			Jejunum Tensile strength (N)
Group^1^		Weight (g)	Breaking strength (N)	Ratio N/g	Weight (g)	Breaking strength (N)	Ratio N/g	
Main effects	Control	17.26	246.52	14.35	10.46	230.24	22.08	6.50^b^
	Halloysite	17.76	246.23	13.95	10.57	258.64	24.79	9.14^a^
	P-value	0.480	0.984	0.619	0.791	0.137	0.130	0.023
	Male	17.38	241.40	13.99	10.15	247.44	24.66	7.74
	Female	17.65	251.36	14.30	10.88	241.44	22.21	7.90
	P-value	0.706	0.503	0.696	0.082	0.757	0.172	0.896
Interaction	C × M	18.04	244.03	13.56	10.89^ab^	242.11	22.38	5.85^*^
	C × F	16.49	249.01	15.14	10.03^bc^	218.37	21.78	9.62^*^
	H × M	16.72	238.76	14.42	9.42^c^	252.76	26.94	7.14
	H × F	18.81	253.70	13.47	11.72^a^	264.52	22.64	8.65
	P-value	0.052	0.909	0.404	< 0.001	0.371	0.150	0.112
	SEM	0.350	7.320	0.393	0.209	9.492	0.890	0.595

^a^^,b^mean values with various letters in the row differ statistically significantly between all groups, considering *P* < 0.05; ^1^C × M, male control group; C × F, female control group; H × M, the experimental male group fed with 1% halloysite; H × F, the experimental female group fed with 1% halloysite; ^*^group C × M was statistically significantly differed from group C × F when NIR test was used (*P* = 0.025)

## Discussion

In our study, the key changes in ducks'production results concerned significant differences in BW at day 42 of rearing and weight gain throughout the rearing period, to the detriment of the halloysite-supplemented groups. Previous studies on broiler chickens and broiler ducks have shown both positive and negative effects of minerals on production results. In the study of Biesek et al. [32], a 4% addition of zeolite to the feed of Pekin broiler ducks significantly reduced the BW of birds during 42 and 49 days of rearing. The authors used Orvia and Cherry Valley duck hybrids and reared with sex division. Still, in the case of origins and sex, there were no significant differences in production results. Chung [49] added a 1% mineral mixture of bentonite and ilite to the Pekin duck's nutrition. The results showed no positive effects on BW, BWG, FI, and FCR. Similar conclusions were presented by Choi [50], who indicates in his research that 1 and 1.5% of ilite did not improve growth performance and economic indicators in broiler duck rearing. According to Chung and Choi [30], using 2% bentonite powder in feed significantly increased the FCR of birds compared to the control group and the group fed with 2% illite powder (*P* = 0.0338).

On the other hand, there are also some studies supporting the beneficial effects of natural minerals on BW, FI, and FCR in broiler chicken rearing [51, 52], which does not correspond to the results of our research. These changes may result from improved digestibility of nutrients from feed [53], changes in jejunal histomorphometry leading to better absorption [54], and modulation of the immune system [55]. In our research, females from the control group were characterized by significantly higher BW and BWG in the first feeding period compared to both groups fed with the addition of halloysite. A significantly higher FCR up to the 28 th day of rearing was found in males fed with halloysite than in the control group. Immediately after hatching, the digestive tract of birds develops dynamically, which is a key moment that can affect the BW of birds [56]. It is suggested that young birds were more sensitive to the halloysite effect in the first rearing period, which translated into changes in the above parameters.

However, no significant changes were found later in the total rearing time. The mechanisms of action of aluminosilicates leading to adverse changes in performance are difficult to identify unambiguously. A key aspect is the origin of minerals, which determines their chemical composition and specific properties [57, 58]. Therefore, there may be large discrepancies in the results of the tests obtained on poultry. Clay minerals are characterized by a high ability to absorb water, contributing to increased intestinal contents viscosity [59]. According to Le Gall-David et al. [60], higher viscosity of intestinal contents in chickens reduces digestive enzymes diffusion and the digestibility of nutrients, which could explain adverse changes in BW and BGW of ducks. In addition, according to Park et al. [61], these changes can also cause a reduction in oxygen levels due to microbial fermentation and an increase in the probability of pathogen invasion, which negatively affects performance. Clay minerals can also absorb proteins of various origins (egg whites, hen egg white lysozyme, bovine serum albumin, α- and β-Lactalbumin) using appropriate mechanisms, including electrostatic and hydrophobic interactions [62]. This could suggest a potentially lower digestibility of feed protein after administration of the halloysite. However, this contradicts the results of the study by Nadziakiewicz et al. [25], who showed that 1% halloysite to broiler chicken feed positively affected the utilization of crude protein.

In our study, halloysite increased the relative liver weight of ducks. Considering the interaction, significantly lower relative liver weight occurred in the control groups compared to drakes fed with the halloysite supplementation. Similarly, Al-Beitawi et al. [63] showed that chickens'liver, heart, and pancreas weights were higher after using 1.5% nan-clay minerals in the feed. This increase may be due to the chemical and physical properties of the minerals. In particular, the content of Na^+^, K^+^, and Ca^2+^ ions may increase the BW and internal organs’ weight.

Many studies have also confirmed the protective effect of clay minerals on ducks'liver after using mycotoxins, thanks to their absorption abilities [64–66]. Li et al. [67] found a decrease in hepatic malondialdehyde activity in young Cherry Valley ducks fed contaminated corn (*P *< 0.05). According to Das et al. [66], bentonite can effectively counteract inflammation of hepatocytes after the action of aflatoxin.

Sex significantly impacted selected characteristics of duck carcasses, as a higher relative gizzard weight was found in males. Considering the interaction, both males in the control group and those fed with halloysite had a higher relative gizzard weight than females in the control group. This corresponds with the results of Wasilewski et al. [68], who confirmed significantly heavier gizzard of male Pekin Cherry Valley SM3 Heavy ducks compared to females (P ≤ 0.05). Kaewtapee et al. [33] also showed a higher gizzard weight in male Cherry Valley ducks. The authors linked this to better growth in males and a higher proportion of pectoral muscles in the carcass, which increased nutrient digestion and absorption. However, it does not explain our results, as males had significantly lower pectoral muscle weight and total muscle than females. At the same time, these changes could translate into a higher weight of male carcass remains. Similarly, a higher weight of carcass residues in males (590.8 g) compared to females (414.0 g) was confirmed by Adamski et al. [69], who analyzed the characteristics of Pekin ducks line P55 carcasses. Previous studies on Pekin ducks have also shown a significantly higher abdominal fat weight of females than males [70, 71], similar to the results of our research. Higher fat deposition in females may be due to the secretion of estrogen, which stimulates the synthesis of yolk lipoproteins, which are not used for egg production and are deposited in the abdominal cavity. This explanation for the changes in abdominal fat content was proposed by Kaewtapee et al. [33] after Cherry Valley ducks 49 days of rearing.

In our research, halloysite increased the pH of the pectoral muscles, collagen, IMF, and water content compared to the control. Similarly, Hcini et al. [72] showed a significantly higher pH of the pectoral muscles of turkeys fed with a 2% addition of zeolite to the feed, indicating that the meat's quality was maintained for a more extended period. According to Safaei et al. [73], the decrease in the pH of meat after slaughter results from the breakdown of glycogen in cells and the production of lactic acid, so it is suggested that halloysite may also have had an impact on this process. Safaei et al. [74] showed a lower IMF content in chicken meat after mixing 1.5% bentonite and kaolin. A similar effect was demonstrated by hydrated aluminosilicates (zeolite and bentonite), which additionally increased the protein content in chicken pectoral muscles (22.69%) compared to the group without minerals in the feed (21.90%) [75]. The chemical composition of pectoral muscles is determined mainly by the energy and protein content of the feed. A high-protein diet promotes higher protein content in the meat [76]. However, muscle protein content is primarily determined genetically [77].

Lipogenesis in birds occurs mainly in the liver, and the IMF content in the pectoral muscles may depend on lipase or adipocyte activity [78]. Therefore, the highest relative liver weight in the halloysite groups could be associated with the highest IMF content in these groups. Pearson's correlation also confirmed that increased protein content in the pectoral muscles translated into decreased collagen, IMF, and water content. The collagen content in the pectoral muscles was positively correlated with salt, IMF, and water content. Research by Xiong et al. [79] also confirmed a negative correlation between fat and protein content in the leg muscles of broiler chickens.

In our study, the leg muscles of ducks in the experimental groups were found to have lower values for a* and b*. Bentonite added to the feed of Japanese quails resulted in higher values for a* in their pectoral muscles, as Gümüş [80] reported. However, Safaei et al. [73] found no effect of bentonite on the color of chicken leg muscles during storage. Meat color is mainly determined by myoglobin, a pigment present in the muscles. The intensity of the redness of muscles can indicate pre-slaughter stress, as less intense color may indicate an anti-stress effect, as Makarski et al. [81] notified. The study by Cheng et al. [82] demonstrated that palygorskite, a type of aluminosilicate, can bind with pigments in the feed and affect the oxidative status of meat.

In the pectoral muscles of females, a significantly higher protein and salt content and a lower IMF content were found compared to males. Similarly, the leg muscles had more collagen and salt and less IMF. Similar relationships were shown by Biesek et al. [83], who analyzed the chemical composition of the meat of Cherry Valley slaughter ducks of SM-3 Heavy hybrids after 49 days of rearing. The pectoral muscles of females were characterized by a significantly higher protein content and a lower IMF content than females. On the other hand, according to Cygan-Szczegielniak and Bogucka [84], the pectoral muscles of male broiler chickens were characterized by a significantly higher protein (*P *= 0.025) and fat (*P* = 0.041) content, which does not correspond to our results. The most straightforward explanation for the changes in the content of individual chemical components of meat depending on sex is the influence of hormones, as Maiorano et al. [85] described, on the example of other species of farm animals. Similar conclusions were presented by Tavaniello et al. [86] on Japanese quails.

Females fed with halloysite supplementation had significantly higher and males had lower femur bone weight compared to females from the control groups. The study by Wegner et al. [87] showed significantly higher greatest length, medial length, greatest breadth of the proximal end, smallest breadth of the corpus, greatest breadth of the distal end, and greatest depth of distal end in males than females of Pekin ducks SM3 and ST5 Heavy from parent flock. The previous study on zeolite in the feeding of broiler ducks had only shown significantly higher tibia bone strength in the male control group and the female experimental group compared to the male experimental group (*P* = 0.048) [88]. Nadziakiewicz et al. [89] found no significant changes in broiler chickens'femur and tibia bone parameters fed with halloysite at 1%. Differences in various authors’ results may be related to the type of mineral, its composition, and environmental factors.

The use of halloysite significantly increased the strength of the jejunum. The control females had higher jejunum tensile strength than the control males. According to Bilgili and Hess [90], the intestines of males had higher tensile strength, as found in hindgut peak force of broiler chickens. However, it does not correspond to the results of our research. Warren and Hamilton [91] draw attention to the role of collagen as a structural protein responsible for tissue tensile strength. The increase in collagen content in the mucosal wall of the small intestine contributes to a greater distance between the capillary wall and the basement membrane of enterocytes, which can significantly hinder the absorption capacity of the intestine. This was confirmed by Arutyunov et al. [92] after examination of humans with chronic heart failure disease. The authors indicated that collagen can be a physical barrier affecting blood microcirculation and nutrient absorption. It is suggested that the significantly higher intestinal tensile strength of ducks fed with 1% halloysite supplementation could be related to the collagen content of the mucosal wall of the jejunum. In addition, it may have also negatively affected BWG in the experimental groups, which has been previously confirmed. On the other hand, the higher jejunum tensile strength prevents broiler carcass contamination of the intestinal content during further processing [90].

## Conclusions

Halloysite negatively affected ducks'weight and weight gain after 42 rearing days. The pectoral muscles (*pectoralis major* and *minor*) of ducks fed this mineral were characterized by a higher pH, a higher IMF content, and a lower protein content. Significant changes, such as redness and yellowness, have been shown in the leg muscles. It can affect the sensory and technological properties of the meat. Additionally, the halloysite beneficially affected the jejunum tensile strength, which is crucial in the safe technological aspect of meat production. Overall, the findings suggest that halloysite can have both positive and negative effects on the production of broiler ducks. More research is needed to fully understand halloysite's potential benefits and limitations in duck feeding.

## Data Availability

No datasets were generated or analysed during the current study.

## Abbreviations

DM: Dry matter
ADF: Acid detergent fiber
NDF: Neutral detergent fiber
ADL: Acid detergent lignin
BW: Body weight
BWG: Body weight gain
FI: Feed intake
FCR: Feed conversion ratio
EPEF: European Production Efficiency Factor
L*: Lightness
a*: Redness
b*: Yellowness
WHC: Water holding capacity
IMF: Intramuscular fat
SEM: Standard error of the mean
C × M: Male control group fed with a commercial diet
C × F: Female control group fed with a commercial diet
H × M: Experimental male group fed with a commercial diet with 1% halloysite supplementation
H × F: Experimental female group fed with a commercial diet with 1% halloysite supplementation
